# Endothelial dysfunction in nonalcoholic steatohepatitis with low cardiac disease risk

**DOI:** 10.1038/s41598-020-65835-y

**Published:** 2020-06-01

**Authors:** Waleed Al-hamoudi, Amani Alsadoon, Mazen Hassanian, Hisham Alkhalidi, Ayman Abdo, Mohamed Nour, Rabih Halwani, Faisal Sanai, Abdulsalam Alsharaabi, Khalid Alswat, Ahmed Hersi, Ali Albenmousa, Faisal Alsaif

**Affiliations:** 1 Department of Medicine, College of Medicine, King Saud University, Riyadh, Saudi Arabia; 2 Liver Research Center, King Saud University, Riyadh, Saudi Arabia; 3 Department of Surgery, College of Medicine, King Saud University, Riyadh, Saudi Arabia; 4 Department of Pathology, College of Medicine, King Saud University, Riyadh, Saudi Arabia; 5 Department of Cardiac Science, College of Medicine, King Saud University, Riyadh, Saudi Arabia; 6 Immunology Research Laboratory and Asthma Research Chair, College of Medicine, King Saud University, Riyadh, Saudi Arabia; 7 Gastroenterology Unit, Department of Medicine, King Abdulaziz Medical City, Jeddah, Saudi Arabia; 8 Department of Liver Transplantation and Hepatobiliary Surgery, King Faisal Specialist Hospital and Research Center, Riyadh, Saudi Arabia

**Keywords:** Non-alcoholic fatty liver disease, Non-alcoholic steatohepatitis

## Abstract

Nonalcoholic fatty liver disease (NAFLD) is the most common liver disease worldwide. We prospectively evaluated endothelial function by assessing flow-mediated dilatation (FMD) of the brachial artery in patients with biopsy-proven NAFLD. This prospective study included 139 patients (50 healthy controls, 47 patients with steatosis and 42 patients with steatohepatitis), all of whom were nondiabetic. Patients with long-standing or uncontrolled hypertension, smokers, and morbidly obese patients were excluded. The medians (ranges) for vascular FMD in the steatohepatitis, steatosis, and control groups were 6% (0–37.5%), 10.8% (0–40%) and 13.6% (0–50%), respectively. The control group had a higher average FMD than the NAFLD group (15.13% vs 10.46%), and statistical significance was reached when the control and steatohepatitis groups were compared (13.6% vs 6%, p = 0.027). Average alanine aminotransferase was significantly higher in the steatohepatitis group than in the steatosis and control groups (54 (U/L) vs 31 (U/L), p = 0.008). Cholesterol levels were similar between all groups. In the multivariate analysis, FMD (OR = 0.85, p = 0.035) and high triglycerides (OR = 76.4, p = 0.009) were significant predictors of steatohepatitis. In the absence of major cardiac risk factors, we demonstrated better endothelial function in healthy controls, evidenced by a higher FMD of the brachial artery than that of patients with steatohepatitis.

## Introduction

Nonalcoholic fatty liver disease (NAFLD) is one of the most common liver diseases worldwide and is the most common cause of abnormal liver enzymes in many developed countries^1–3^. NAFLD is considered to be a component of metabolic syndrome.

Insulin resistance (a proatherogenic factor) plays a major role in the pathogenesis of various components of metabolic syndrome, including obesity, diabetes mellitus (DM), dyslipidemia and NAFLD^4^. Evidence supporting the relationship between NAFLD and cardiovascular disease (CVD) has increased significantly over the past few years. For example, several epidemiological studies have indicated that NAFLD is linked to an increased risk of CVD^5,6^. This suggests that NAFLD is not just an unrelated comorbidity but that it may also be actively involved in the pathogenesis of CVD. The liver releases a number of mediators, including C-reactive protein, plasminogen, fibrinogen and other inflammatory cytokines that are considered proatherogenic, which may link NAFLD to the pathogenesis of CVD and endothelial dysfunction^7^, and recently, endothelial dysfunction has been described in patients with fatty liver. The majority of studies evaluating the link between NAFLD and atherosclerosis have used ultrasound as the tool for diagnosing NAFLD. Although ultrasound is a good diagnostic tool, its sensitivity for diagnosing mild steatosis is poor; furthermore, ultrasound cannot differentiate simple steatosis from steatohepatitis^8,9^. This study was designed to assess endothelial function in patients with biopsy-proven NAFLD and low cardiovascular (CV) risk by assessing the flow-mediated dilatation (FMD) of the brachial artery. The endothelium is a monolayer of cells that covers the inner surfaces of blood vessels. The endothelium has a wide range of functions and has anti-inflammatory and antithrombotic roles, among others. The endothelium also regulates the diameters of blood vessels by releasing multiple vascular mediators that directly affect vascular tone^10^. FMD of the brachial artery is the most commonly used method to evaluate endothelial dysfunction. The FMD assessment has the advantages of being noninvasive, reproducible, and accurate. With the high prevalence of NAFLD, the association between NAFLD and CVD needs to be studied more thoroughly. This relationship may have future implications for screening and the implementation of early treatment strategies to reduce the risk of CVD in patients with NAFLD. The aim of this study was to assess and compare the endothelial function of the brachial artery in NAFLD patients and healthy control participants.

## Materials and Methods

### Study population

Patients were recruited at the time of admission for laparoscopic cholecystectomy at King Khalid University Hospital (KKUH), Riyadh, from January 2012 to May 2016. This study was conducted in accordance with the Declaration of Helsinki and was approved by the institutional review board of King Saud University Hospital (IRB No. E-11–442). In addition, informed consent was obtained from each participant. Patients were excluded if they met any of the following criteria:^1^ history of alcohol intake;^2^ chronic liver disease of any other etiology;^3^ CVD;^4^ DM;^5^ neurological disease;^6^ chronic hypertension (>5 years), uncontrolled hypertension, or use of more than 2 antihypertensive dugs;^7^ morbid obesity (body mass index (BMI) > 40);^8^ smoking;^9^ cirrhosis or evidence of portal hypertension, laboratory findings of a platelet count <150 (109/L), or an international normalized ratio (INR) ≥ 1.3; or^10^ chronic kidney disease (glomerular filtration rate (GFR) < 60).

### Methods

Patient consent to participate in the study was obtained during the first visit to the clinic, and the following panel of laboratory tests was requested: a complete blood count; serum concentrations of alanine aminotransferase (ALT), aspartate aminotransferase (AST), alkaline phosphatase (ALP), total bilirubin, albumin, fasting glucose, and hemoglobin a1c (HbA1c); and the lipid profile. Serology for hepatitis B and hepatitis C was also included in the initial assessment. Additionally, all participants received a complete assessment of metabolic and autoimmune liver disease. All laboratory tests were performed at the central laboratory of our institution.

Brachial artery FMD testing was conducted at our vascular laboratory by a single expert vascular technician to minimize operator-related biases. To minimize other limitations, the FMD of all subjects was evaluated in a preselected, quiet and temperature-controlled room. A standard protocol was designed prior to the study and involved placing a blood pressure cuff 2 cm below the antecubital fossa and inflating it to approximately 200 mmHg for a total of 3 minutes. Doppler images were acquired at baseline, during occlusion, and three minutes postocclusion. FMD was calculated as the average of the postocclusion dilatation (the postocclusion diameter minus the baseline diameter was divided by the baseline diameter, and the result was multiplied by 100). This protocol did not include the use of nitroglycerin to assess endothelial-independent dilatation.

**Figure 1:**
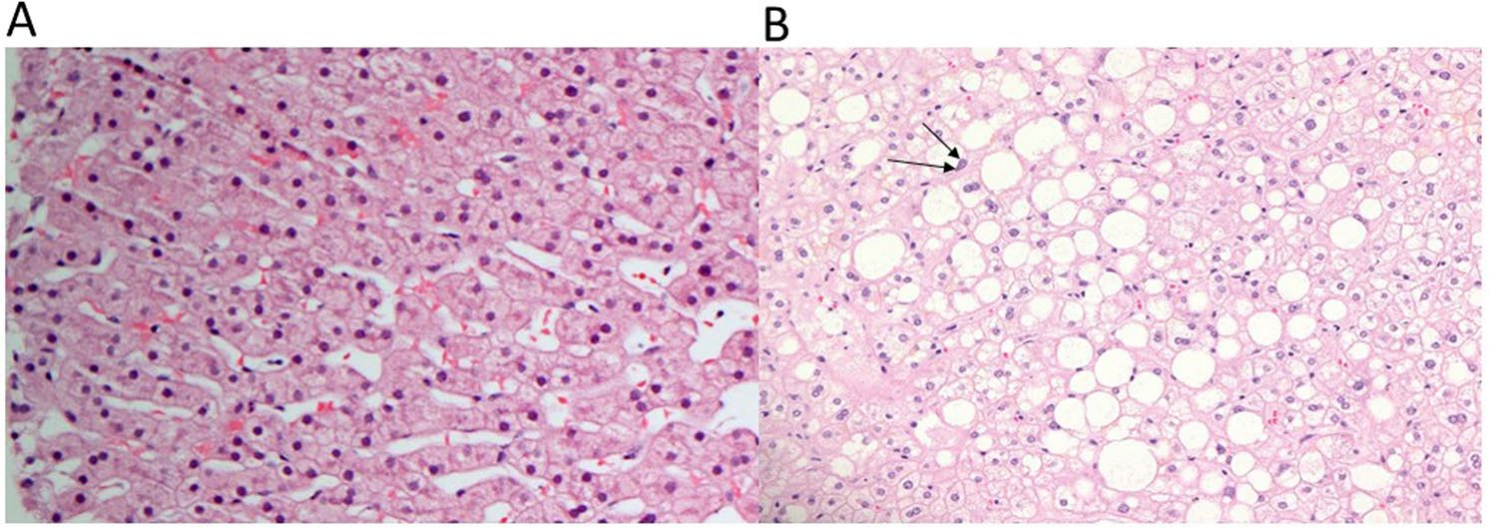
(**A**) Normal liver histology. **(B**) Macrovesicular steatosis with large fat droplets displacing the nucleus to the periphery in the majority of cells (arrows). (Hematoxylin and eosin stain, magnification ×200).

All liver biopsies were taken at the same time as cholecystectomy using a biopsy gun (18 × 20 cm BARD, Max-Core, Arizona, USA) from the right lobe. To minimize any effect of surgery on the hepatic tissue all liver biopsies were obtained prior to any surgical manipulation of the liver. This will also allow the surgeon to observe the biopsy site during surgery for any potential bleed. Biopsies were placed in 10% formalin, transferred to the Histopathology Laboratory and stained using routine hematoxylin and eosin staining (Fig. 1). All biopsies were reviewed and interpreted by an experienced hepatopathologist. The grading percentages for steatosis were as follows: grade 0, <5%; grade 1, 5–33%; grade 2, >33–66%; and grade 3, >66%. Normal findings were considered grade 0 for steatosis and stage 0–1 for fibrosis with no or mild inflammation. Fatty liver was defined as the presence of steatotic hepatocytes at a proportion of at least 5% with or without mild lobular or portal inflammation and stage 0–1 fibrosis. All liver biopsies contained at least 10 well-preserved portal tracts and were considered adequate upon pathological analysis.

The histological diagnosis of nonalcoholic steatohepatitis (NASH) was based on NAFLD activity score, which combined the steatosis grade, hepatocyte ballooning and lobular inflammation with or without fibrosis; a score of 5 or more indicated NASH. Both the vascular technician and pathologist were blinded to all clinical information.

### Statistical analyses

Descriptive and inferential analyses were performed using SPSS 17. Categorical data are described as frequencies and percentages and were compared using the chi-square or Fisher’s exact test, whichever was appropriate. Numerical data are described as the means and standard deviations and were compared using Student’s t test for normally distributed variables or as medians and ranges if they were not normally distributed. The Mann-Whitney test was used to compare 2 nonparametric samples, and the Kruskal-Wallis test was used for more than 2 samples. Differences were considered significant if the p value was <0.05. We evaluated the performance of FMD in the identification of patients with NASH using receiver operating characteristic (ROC) curves.

## Results

**Figure 2:**
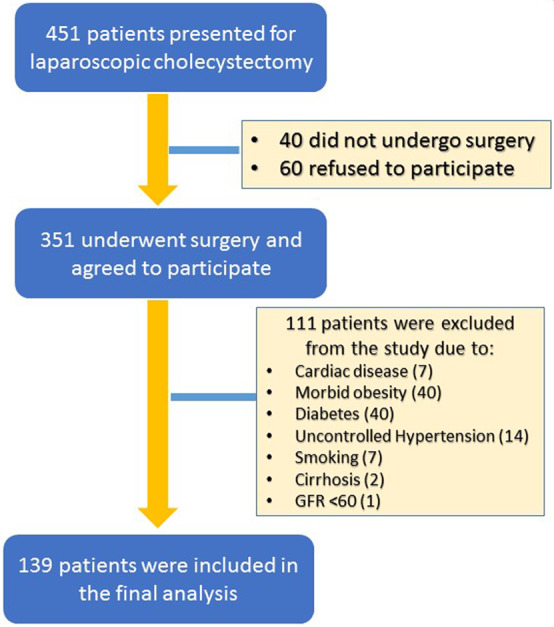
Flow chart of patients who met the inclusion/exclusion criteria for the study.

A total of 139 patients (34 males and 105 females) were included in this prospective study (50 healthy controls, 47 NAFLD patients with simple steatosis, and 42 NAFLD patients with steatohepatitis) (Table 1). The average ages in the control, steatosis and steatohepatitis groups were 35.1 ± 13, 41.0 ± 8.7 and 46.0 ± 9.6 years, respectively. The average BMIs of the three groups were 29.5 ± 4.8, 32.0 ± 5.2, and 31.5 ± 5.5, respectively. A total of 351 patients provided consent and were enrolled during the initial stage of recruitment. All included individuals had no history of diabetes, and their HbA1c values were <6.4. Forty patients were excluded because of either an established diagnosis of diabetes or an HbA1c of more than 6.4 at the time of presentation. Furthermore, patients with chronic hypertension or uncontrolled blood pressure were excluded (all individuals had a blood pressure <150/90 prior to FMD assessments). Four patients were on multiple antihypertensive medications and were therefore excluded; two other patients were excluded because of high blood pressure prior to FMD assessments. Eight patients had an established diagnosis of hypertension for at least 5 years and were excluded. Forty patients who were morbidly obese (BMI > 40) were also excluded (Fig. 2). Because of the high prevalence of metabolic syndrome in our NAFLD population, the study was extended to complete recruitment for the steatohepatitis group. No major bleeding or other significant surgical complications were encountered during laparoscopic liver biopsy.

**Table 1:** Characteristics of patients in the three groups.

Mean & standard deviation or median & range	NASH group N = 42	Steatosis group N = 47	Control group N = 50	P value NASH vs control	P value steatosis vs control	P value steatosis vs NASH
Male/female	12/30	13/34	9/41	0.210	0.213	0.974
Age	46.9 ± 9.6	41.0 ± 8.7	35.1 ± 13.0	0.000	0.012	0.003
BMI (kg/m^2^)	31.5 ± 5.5	32.0 ± 5.2	29.5 ± 4.8	0.88	0.019	0.657
Hemoglobin (g/l)	129 (108–158)	132 (78–174)	130 (97–166)	0.485	0.467	0.995
Platelets (10^9^/L)	266 (204–426)	276 (179–746)	284 (141–475)	0.237	1.000	0.213
ALT (U/L)	34 (17–127)	37 (12–76)	31 (19–112)	0.005	0.453	0.053
Albumin (mg/L)	37.5 (29–44)	38 (30–45)	37 (27–44)	0.444	0.801	0.341
Bilirubin (µmol/L)	7.5 (2.5–38)	6 (2–18)	6.2 (2–15)	0.271	0.567	0.156
INR	1 (0.9–1.2)	1 (0.9–1.1)	1 (0.9–1.2)	0.282	0.372	0.125
Ferritin (µg/l)	60.2 (4.6–263)	51 (6–364)	25.4 (8–348)	0.007	0.014	0.767
Cholesterol (mmol/L)	5.2 (3.4–9.9)	4.9 (3–7.3)	4.8 (3–7.4)	0.239	0.168	0.705
Triglycerides (mmol/L)	1.5 (0.6–8.0)	1.2 (0.7–3.1)	0.9 (0.2–2.7)	0.002	0.001	0.885
Flow-mediated dilatation (%)	6% (0–38)	9.3 (0–40)	13.6% (0–50)	0.027	0.145	0.322

A summary of the baseline characteristics of the three groups is provided in Table 1. The high prevalence of metabolic syndrome resulted in the exclusion of a significant number of potential participants; however, the first consecutive target population that met the inclusion criteria in each group was recruited (Fig. 2). The medians and ranges for vascular FMD in the NASH, steatosis, and control groups were 6% (0–37.5%), 10.8% (0–40%) and 13.6% (0–50%), respectively. The patients in the control group had a higher average FMD than the patients with NAFLD (15.13 vs 10.46%), and the difference between the control group and the NASH group was significant (13.6% vs 6%, p = 0.027). The average ALT level was significantly higher in the NASH group than in the steatosis and control groups (54 vs 31, p = 0.008).

Ferritin and triglyceride levels were similar between patients with NASH and those with steatosis but were significantly higher in these groups than in the control group (p = 0.002). Additionally, the platelet counts were similar between all three groups; this was an expected finding, as none of the patients in this study had evidence of portal hypertension. Additionally, the albumin, bilirubin and INR levels were similar between all three groups, indicating that liver function was preserved. Two patients with biopsy-proven NASH-related liver cirrhosis were excluded from this study. In the multivariate analysis using binary logistic regression, FMD (OR = 0.85, p = 0.041) and TG (OR = 76.4, p = 0.009) were predictors of NASH compared to the control group (Tables 2–4).

**Table 2:** Comparison of the NAFLD and control groups (multivariate analysis).

	OR	P value	95% CI
Age	1.031	0.354	0.967–1.100
Sex	1.149	0.934	0.044–29.893
BMI (kg/m^2^)	1.032	0.644	0.903–1.178
Hemoglobin (g/l)	0.957	0.210	0.894–1.025
Platelets (10^9^/L)	1.000	0.955	0.993–1.007
ALT (U/L)	1.019	0.445	0.971–1.068
Albumin (mg/L)	1.060	0.621	0.840–1.338
Bilirubin (µmol/L)	0.872	0.108	0.738–1.031
Ferritin (µg/l)	1.012	0.104	0.997–1.027
Cholesterol (mmol/L)	0.872	0.723	0.408–1.862
Triglycerides(mmol/L)	7.725	0.003	1.547–38.562
Flow-mediated dilatation (%)	0.948	0.111	0.888–1.012

**Table 3:** Comparison of the steatosis and control groups (multivariate analysis).

	OR	P value	95% CI
Age	1.006	0.883	0.928–1.090
Sex	0.074	0.280	0.001–8.304
BMI (kg/m^2^)	1.072	0.406	0.910–1.264
Hemoglobin (g/l)	0.903	0.061	0.811–1.005
Platelets (10^9^/L)	1.003	0.515	0.995–1.011
ALT (U/L)	0.992	0.779	0.934–1.052
Albumin (mg/L)	1.067	0.648	0.806–1.413
Bilirubin (µmol/L)	0.708	0.060	0.494–1.014
Ferritin (µg/l)	1.018	0.035	1.001–1.035
Cholesterol (mmol/L)	0.806	0.648	0.319–2.034
Triglycerides (mmol/L)	11.369	0.021	1.449–89.224
Flow-mediated dilatation (%)	0.955	0.248	0.883–1.033

**Table 4:** Predictors of NASH in the multivariate analysis compared to the control group/.

	OR	P value	95% CI
Age	1.165	0.085	0.979–1.385
Sex	11.152	0.443	0.024–5283.388
BMI (kg/m^2^)	0.829	0.208	0.618–1.110
Hemoglobin (g/l)	0.903	0.316	0.740–1.102
Platelets (10^9^/L)	0.991	0.363	0.972–1.011
ALT (U/L)	1.089	0.100	0.984–1.205
Albumin (mg/L)	1.140	0.583	0.713–1.823
Bilirubin (µmol/L)	0.820	0.269	0.578–1.165
Ferritin (µg/l)	1.017	0.281	0.986–1.049
Cholesterol (mmol/L)	0.542	0.461	0.106–2.768
Triglycerides (mmol/L)	76.397	0.009	0.000–0.309
Flow-mediated dilatation (%)	0.851	0.041	0.730–0.993

**Figure 3:**
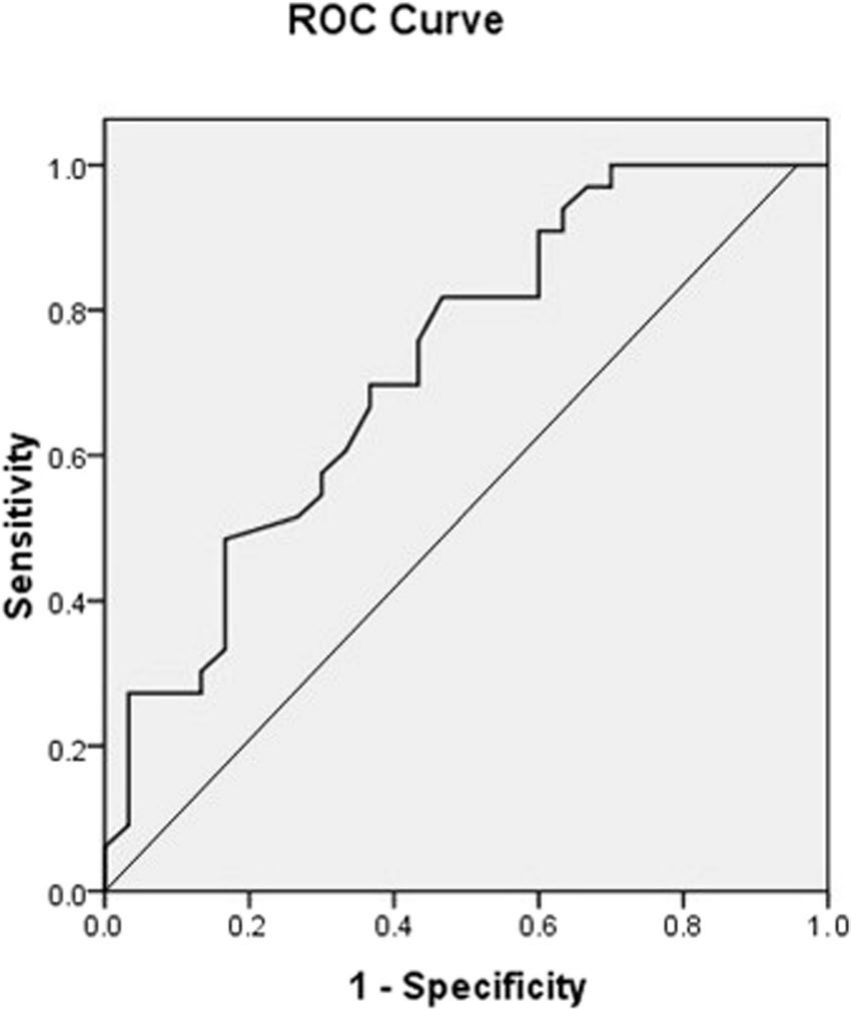
Receiver operating characteristic curve of sensitivity (true-positive fraction) plotted against 1-specificity (false-positive fraction) in steatohepatitis patients.

We evaluated the performance of FMD in the identification of patients with NASH. The area under the ROC curve was 0.725 (95% CI 0.600–0.850, p = 0.002). An FMD cutoff value of 11.35% had a sensitivity of 70% and specificity of 63.3%. Figure 3 shows the ROC curve.

## Discussion

To the best of our knowledge, this is the only study assessing endothelial function in the absence of diabetes, morbid obesity, smoking and poorly controlled hypertension in patients with biopsy-proven NAFLD.

NAFLD is a group of disorders that occur in people who do not drink excessive amounts of alcohol and that are characterized by abnormal fat accumulation in the liver, leading to histological features that are similar to those seen in alcoholic liver disease^11^.

This clinical entity is comprised of a spectrum that begins with simple fatty liver (steatosis) and may end with cirrhosis. A high prevalence of ultrasonographic findings of fatty liver and insulin resistance has been reported in patients with CVD^12^.

In the current study, we evaluated endothelial function in patients with conditions ranging from normal liver histology to NASH. We also eliminated factors that are clearly associated with endothelial dysfunction and CVD, including diabetes, smoking, chronic or uncontrolled hypertension, known cardiac disease and liver cirrhosis. It can be argued that the increased atherosclerosis and CV events in NAFLD patients are related to the associated metabolic syndrome, especially diabetes. It was recently shown that patients with type 2 diabetes and ultrasonographically diagnosed NAFLD had higher rates of coronary, cerebrovascular and peripheral vascular disease than age‐ and sex‐matched patients with diabetes without NAFLD^6^. We did not exclude patients with hyperlipidemia; however, cholesterol levels were not elevated in the majority of our study population and were similar between all three groups. It has been well established that vascular dysfunction can be induced by smoking. Smoking-induced vascular dysfunction is initiated by reduced nitric oxide (NO) bioavailability and the increased expression of adhesion molecules, leading to subsequent endothelial dysfunction. Smoking also increases platelet and macrophage adherence and promotes the development of a procoagulant and inflammatory environment^13^. The endothelial response to various agents and physical stimuli is impaired in patients with hypertension. The decline in endothelial function in systemic hypertension is associated with a progressive decrease in NO bioavailability and an increase in the production of endothelium-derived contraction factors^14^. We did not completely exclude hypertensive patients in the current study; however, we included patients with only a relatively short-term history of hypertension, who were on one or two antihypertensive agents and whose hypertension was well controlled. Endothelial dysfunction was also reported in patients with cirrhosis and advanced liver disease. Multiple studies have clearly shown systemic vasodilation in patients with cirrhosis; NO is overproduced in cirrhosis, and the measured serum levels are significantly elevated in both patients with cirrhosis and animal models of cirrhosis^15–17^. NO is an endothelial-derived relaxing factor that leads to arterial vasodilatation. Therefore, patients with advanced liver disease were also excluded.

Saudi Arabia is among the countries with the highest prevalence of metabolic syndrome, DM, and obesity^18–21^. This made the recruitment of patients difficult and prolonged, but it also contributed to a more accurate assessment by eliminating the negative impact of metabolic syndrome on endothelial function.

Brachial FMD is the most common tool used to assess endothelial function in the majority of clinical studies^22–24^. It is a noninvasive and reproducible method with relatively accurate results. Therefore, we used FMD to evaluate endothelial function in the current study, and this method enabled us to compare our results with those of other clinical trials. Our vascular evaluation protocol was designed to minimize limitations; thus, the test was performed by a single well-trained and experienced technician in a quiet and temperature-controlled room. In our study, we clearly demonstrated that there was a higher vasodilatory response in the patients of the control group than in the NAFLD patients. This difference was more evident when the control and steatohepatitis groups were compared. The lower endothelial flow‐mediated vasodilation is likely a reflection of the presence of subclinical atherosclerosis. Similarly, a relationship between NAFLD and atherosclerosis has been reported. Fracanzani and colleagues demonstrated that patients with NAFLD had a thicker carotid intima-media and a higher prevalence of plaques than healthy controls. Steatosis was the strongest variable to be independently associated with intima-media thickness and the development of atherosclerosis^25^. Villanova and colleagues demonstrated that there was evidence of endothelial dysfunction and an increased risk of CV events in NAFLD. They measured the vasodilatory response of the brachial artery in response to ischemia as well as the CV risk profile in 52 NAFLD patients and 28 age- and sex-matched controls. They demonstrated that there was evidence of endothelial dysfunction and an increased risk of CV events in NAFLD patients compared to healthy controls. However, the diagnosis of NAFLD in their study was based on chronically raised alanine aminotransferase levels and a bright liver on ultrasound. Furthermore, they excluded only patients with overt diabetes or a BMI above 35 kg/m^2^^26^. Senturk and colleagues showed that patients with NASH had significantly worse endothelial dysfunction than patients with simple steatosis^27^. Endothelial dysfunction is considered to be a strong predictor for the development of atherosclerosis and CVD. Shechter *et al*. conducted a study to determine the long-term association between brachial artery FMD and adverse CV events in healthy subjects, and they prospectively assessed brachial FMD data from 618 consecutive healthy subjects with no apparent heart disease. The subjects were divided into 2 groups, namely, FMD < 11.3% and >11.3%, where 11.3% was the median FMD, and the patients were comparable regarding other CV risk factors. During a mean clinical follow-up of 4.6 years, composite CV events were significantly more common in subjects with an FMD < 11.3% than in subjects with an FMD > 11.3% (15.2% vs 1.2%, p < 0.0001, respectively). Univariate and multivariate analyses demonstrated that the median FMD was the best independent predictor of long-term CV adverse events (odds ratio 2.93, 95% confidence interval (CI) 1.28 to 6.68, p < 0.001)^28^.

One of the limitations of this study is the relatively small sample size in each group. The lack of serum biological markers of endothelial dysfunction in the current study was another limitation. Nevertheless, this study clearly revealed a variable response in brachial artery FMD across the spectrum of NAFLD.

## Conclusion

We demonstrated that in the absence of diabetes, morbid obesity and uncontrolled hypertension, healthy controls have better endothelial function than biopsy-proven NAFLD patients, as shown by a higher FMD of the brachial artery. This finding may be related to the higher incidence of CV events in NAFLD patients. The assessment of endothelial function through the evaluation of the FMD of the brachial artery has been shown to be an accurate, reproducible and noninvasive method that may have a potential role in the CV risk stratification of patients with NAFLD.

## Data Availability

The data that support the findings of this study are available from the authors upon request.
